# Assimilation of virtual legs and perception of floor texture by complete paraplegic patients receiving artificial tactile feedback

**DOI:** 10.1038/srep32293

**Published:** 2016-09-19

**Authors:** Solaiman Shokur, Simone Gallo, Renan C. Moioli, Ana Rita C. Donati, Edgard Morya, Hannes Bleuler, Miguel A.L. Nicolelis

**Affiliations:** 1 Neurorehabilitation Laboratory, Associação Alberto Santos Dumont para Apoio à Pesquisa (AASDAP), São Paulo, Brazil; 2 STI IMT, Ecole Polytechnique Fédérale de Lausanne, Lausanne, Switzerland; 3 Edmond and Lily Safra International Institute of Neuroscience, Santos Dumont Institute, Macaiba, Brazil; 4 Alberto Santos Dumont Education and Research Institute, São Paulo, Brazil; 5 Associação de Assistência à Criança Deficiente (AACD), São Paulo, Brazil; 6 Department of Neurobiology, Duke University, Durham, NC, USA; 7 Department of Biomedical Engineering, Duke University, Durham, NC, USA; 8 Department of Psychology and Neuroscience, Duke University, Durham, NC, USA; 9 Center for Neuroengineering, Duke University, Durham, NC, USA

## Abstract

Spinal cord injuries disrupt bidirectional communication between the patient’s brain and body. Here, we demonstrate a new approach for reproducing lower limb somatosensory feedback in paraplegics by remapping missing leg/foot tactile sensations onto the skin of patients’ forearms. A portable haptic display was tested in eight patients in a setup where the lower limbs were simulated using immersive virtual reality (VR). For six out of eight patients, the haptic display induced the realistic illusion of walking on three different types of floor surfaces: beach sand, a paved street or grass. Additionally, patients experienced the movements of the virtual legs during the swing phase or the sensation of the foot rolling on the floor while walking. Relying solely on this tactile feedback, patients reported the position of the avatar leg during virtual walking. Crossmodal interference between vision of the virtual legs and tactile feedback revealed that patients assimilated the virtual lower limbs as if they were their own legs. We propose that the addition of tactile feedback to neuroprosthetic devices is essential to restore a full lower limb perceptual experience in spinal cord injury (SCI) patients, and will ultimately, lead to a higher rate of prosthetic acceptance/use and a better level of motor proficiency.

Spinal cord injuries can induce significant bidirectional loss of communication between the subject’s brain and his/her body, i.e. the patient can neither generate body movements as a result of the loss of cortical communication with the spinal cord, nor can their brain receive somatosensory feedback originating in the body’s periphery^1^. For the past 15 years, studies in animals^2^,^3^ and patients^4^ have suggested that brain-machine interfaces (BMI) may provide a novel therapeutic alternative to restore mobility in severely paralyzed patients^5^. Yet, despite the high prevalence of concurrent somatosensory and motor deficits in such patients, few BMI studies have aimed at restoring both motor and somatosensory feedback simultaneously in paralyzed subjects^3^,^6^.

In the absence of tactile feedback, BMI users have to rely mainly on vision to enact their direct brain control of an artificial actuator. Since in these cases the user does not receive any tactile or proprioceptive feedback related to the actuator performance, we and others have argued that such BMI setups are unlikely to have a significant clinical impact on severely impaired patients^4^. This is easy to understand when one realizes that a patient would have to look at his prosthetic hand every time he moves it to reach and hold an object, or look at the floor every time his lower limb exoskeleton touches the ground during autonomous locomotion.

As part of a project to develop and test a non-invasive, brain-controlled lower body exoskeleton for SCI patients, we implemented a new rehabilitation paradigm in which patients interacted with a rich immersive virtual reality environment^7^ (VRE). In this VRE, patients interacted with the movements of 3D virtual avatar legs on different surfaces and environments, through a virtual reality head mounted display (Fig. 1a). During this task, patients also wore a haptic display device integrated in the long sleeves of a shirt (Fig. 1a). Each of the sleeves contained three small coin-shaped vibrators lined up along the forearm’s distal-proximal axis. By varying the magnitude and temporal sequence of activation of these vibrators, we were able to deliver somatosensory information, originating from the movements of virtual limbs observed by the eight SCI patients, onto the skin surface of their forearms.. Our central goal was to allow these SCI patients to use this haptic display in order to sense the position of the virtual legs in space, the contact of the virtual foot with the floor, and the type of surface with which they were in contact. In this context, the tactile paradigm presented here is at the cross-roads of two major concepts: sensory substitution and sensory remapping. Sensory substitution consists of using one sensory modality to carry information from another one^8^. This can be seen in the classic studies of Bach-y-Rita in which visually impaired patients learned to use a matrix of vibrators to translate visual information into tactile feedback delivered on the skin of the back^8^ or on the tongue’s surface^9^. Haptic displays have also been used to mitigate audition^10^ or vestibular deficits^11^,^12^ among others. On the other hand, sensory remapping techniques (for example those used with amputee patients) aim at providing somatotopically matching haptic stimulation onto a different body part^13^.

Here, we implemented a paradigm that includes: (a) a non-invasive tactile remapping, obtained by providing vibrotactile^14^ stimulation on the patient’s forearms to inform about lower limb tactile and proprioceptive information during a virtual gait/walking task; and (b) a tactile substitution procedure in which complex haptic cues (tactile and proprioceptive information from lower limbs) were replaced with simpler encoded tactile stimuli.

Using this apparatus, SCI patients were able to receive both the tactile feedback providing temporal and physical cues about virtual feet interacting with different floor surfaces, and the proprioceptive feedback describing the position in space of virtual lower limbs. Analysis of the patients’ performance in a crossmodal congruency task^15^ revealed that subjects experienced an assimilation of the virtual legs as a projection of their own body.

## Results

**Figure 1:**
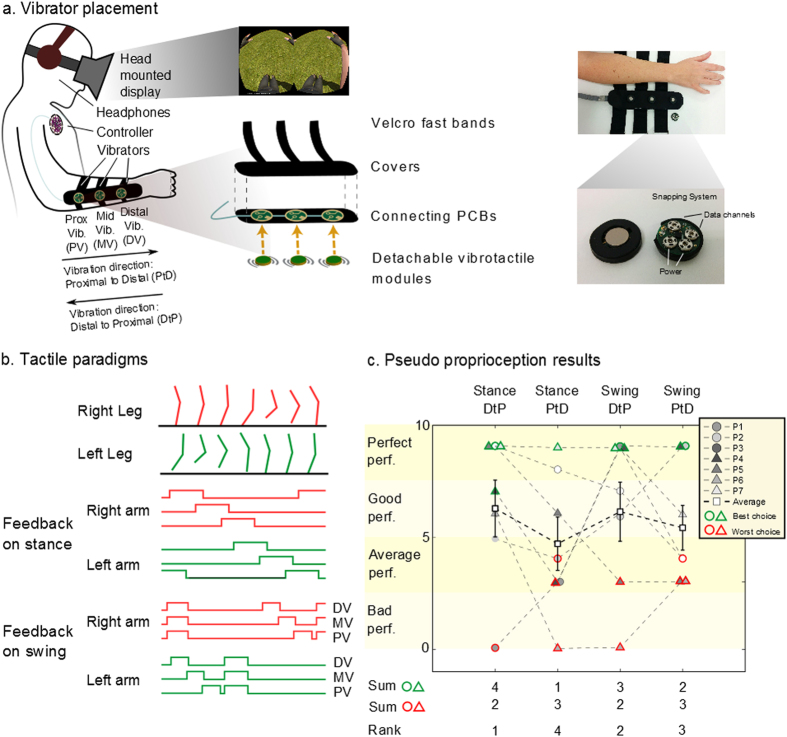
Tactile shirt and pseudo proprioception results. (**a**) Subject wearing the tactile shirt. Three vibrators are aligned on ulna bone. Detachable vibrators are connected to a PCB converted by cloth and Velcro bands. Details of the vibrator modules are shown: the clipping systems are used both as mechanical clipping and electrical connectors (power + data). (**b**) Two different tactile paradigms are proposed: feedback on stance (tactile feedback rolls on the forearm of the patient in synchrony with the foot contact) or swing (feedback roll during the swing and all three vibrators at once on foot contact). (**c**) Results given for the experiment evaluating the pseudo proprioception test with four different tactile feedbacks (left to right): 1) feedback on stance moving from wrist to elbow (Distal to Proximal, DtP), 2) feedback on stance moving from elbow to wrist (Proximal to Distal, PtD), 3) on swing DtP and 4) on swing PtD. Patients used their arms to show the position of the walking avatar’s legs, relying on tactile feedback only. Scores based on ability to reproduce the avatar walk for different speeds are reported including the number of patients for whom a certain paradigm was the best or the worst choice and the rank of the four paradigms.

All experiments were performed with naïve SCI patients (Table S1) using an immersive virtual reality system where the subjects’ lower limbs were simulated by a human-like 3D avatar seen from the first person’s perspective through a head mounted display (Fig. 1a). Tactile and proprioceptive sensations generated by the avatar’s virtual legs were mapped on the patients’ forearms by means of arrays of vibrators that defined a haptic display (Fig. 1a). Initially, we were interested in identifying the most intuitive tactile feedback paradigm for SCI patients to perceive the position of their legs when relying on tactile feedback only. Next, we analyzed how the avatar legs were assimilated into the patients’ brain body representation using the same apparatus. Lastly, we investigated the parameters of tactile stimulation that allowed our patients to experience the vivid sensation of walking on three different types of ground surface: sand, grass or paved street.

### An Intuitive Representation of Leg Position Using a Forearm Haptic Display

We ran experiments with seven (P1-P7) patients. Here, we considered different tactile feedback modalities and searched for the one that patients felt was more intuitive for perceiving the position of the avatar leg when relying on the tactile feedback solely. Two main questions were asked: (a) Should the artificial feedback represent the rolling of the foot on the floor (feedback on stance, Fig. 1b) or the swinging of the leg when the body is balancing; and (b) Should the tactile stimulation be delivered from the wrist to the elbow (Distal to Proximal, DtP) or from the elbow to the wrist (Proximal to Distal, PtD). This was important as the artificial tactile feedback proposed here is an abstract substitution of the sensory feedback from the legs. Thus, the direction of the movement on the forearm can be perceived as representing different phases of the gait cycle. For example, during the stance phase, a PtD sequence of forearm vibration reproduces the same direction of movement produced by the foot rolling on the floor (i.e. perception of the foot going forward). Meanwhile, a DtP sequence moves in the same direction as the foot’s movement in relation to the subject’s body (perception of the body going forward compared to the foot during stance).

To induce a movement sensation on the forearm while using an array of eccentric rotating mass (ERM) vibrators, we employed a well-documented illusion called the tactile apparent movement^16^,^17^. This illusion appears when two vibrating actuators are sequentially triggered on the subject’s skin with a specific stimulation length and specific delay between the onset of the two vibrations. As a result, the subject perceives one continuous touch going from the first to second vibrator instead of two discrete stimuli. The parameters of stimulation length and onset delay inducing the apparent movement illusion were calculated for each patient (see Supplementary Methods, Fig. S1).

During the experiment, patients were asked to report the position of the avatar leg relying on tactile feedback only. The avatar’s walk was randomly changed between three speeds (Fig. S2a). A score, ranging from 0 to 3, was given to evaluate a patient’s performance for each speed: 0 - if the patient could not report the position of the avatar leg; 1 - if patients could report the position at constant speed; 2 - if they could report the position when the speed was changed, but confused the left and right leg; and 3 - for a perfect execution of the task. Final score was the sum of scores for the three speeds and thus ranged between 0 and 9.

Three out of seven patients accurately reported the leg position at every speed when the tactile feedback was of type Stance-Distal to Proximal (DtP), three had good performance (score range 5–8), and one was unable to report the leg position (Fig. 1c, Table S2 for details). For the Stance-Proximal to Distal (PtD) paradigm, three patients had good or perfect performances whereas the other four had average to bad performance (score range 1–4). Three patients were perfect with the Swing-Distal to Proximal (DtP) tactile paradigm, two were good and the last two were average or bad. Finally, for Swing-Proximal to Distal (PtD) paradigm, four patients exhibited average performance, one was good and two were perfect. We observed that four patients had their highest score with Stance-DtP, one reached maximal performance with Stance-PtD, three with Swing-DtP and two with Swing-PtD.

Not every patient had his/her best performance with the same tactile paradigm. However, we wanted to use only one type of tactile feedback for the whole group for all tests with the tactile shirt. Therefore, we identified the type of feedback that elicited the best performance in a majority of our subjects. Using a simple metric ranking (see Methods), we classified the four paradigms as follows (from best to worst): Stance-DtP, Swing-DtP, Swing-PtD and Stance-PtD (Fig. 1c). Overall, tactile stimulation going from Distal to Proximal was more intuitive for feedback delivered during both the avatar leg Stance and Swing phases. At the end of the experiment, patients were also asked to report their subjective experience (Fig. S2b). All patients answered that they ‘agreed’ or ‘totally agreed’ that ‘the tactile feedback [matched] the walk’ when the tactile feedback was on Stance-DtP, Swing-DtP, and Swing-PtD. For Stance-PtD, one patient ‘disagreed’ that feedback matched the walk. No patient reported that ‘The tactile feedback was disturbing’ for any of the four feedback modalities. All patients ‘agreed’ or ‘totally agreed’ they ‘could imagine/feel [themselves] walking well with [their] eyes closed, and [they] knew where the avatar legs were’ for Stance-DtP, Swing-DtP, and Swing-PtD, while one patient disagreed with that statement during the Stance-PtD modality (Fig. S2b).

Considering both the behavioral assessment and the responses to the questionnaire, it appeared that feedback on Stance, with a sequence of vibrations going from wrist to elbow (DtP) was the best feedback at the group level to deliver tactile feedback to our patients using our forearm haptic display. Note that during the experiments patients were never told what the vibrotactile pattern represented (if it was on Stance or on Swing, etc.). This allowed them to make bias-free connections between the vibrotactile stimulation and the avatar’s walk.

### Incorporation of Virtual Legs with Visuo-Tactile Stimulation

After we identified the most intuitive protocol for delivering tactile feedback, we investigated the change in the patients’ perception of the avatar legs in relation to their own bodies while using our haptic display. We used a psychophysical measurement while tactile feedback (of type Stance-DtP) was synchronized with the avatar walk (Vision + Tactile, VT). We compared this condition to the case where tactile feedback was absent and only visual feedback was provided (Vision only, VO).

We employed the Crossmodal Congruency Task (CCT)^15^,^18^,^19^, a well-documented indirect measurement of tool incorporation by human subjects, and the corresponding Cross Congruent Effect (CCE), which calculates the interaction between a vibration on the body and a visual distractor on the tip of the tool (in our case the avatar feet), to measure how the virtual avatar legs were assimilated by our SCI patients (high CCE correlates with integration of the tool; see Experimental Procedures). All our patients were able to discriminate the position of the vibrators used in this experiment (Fig. S3).

**Figure 2:**
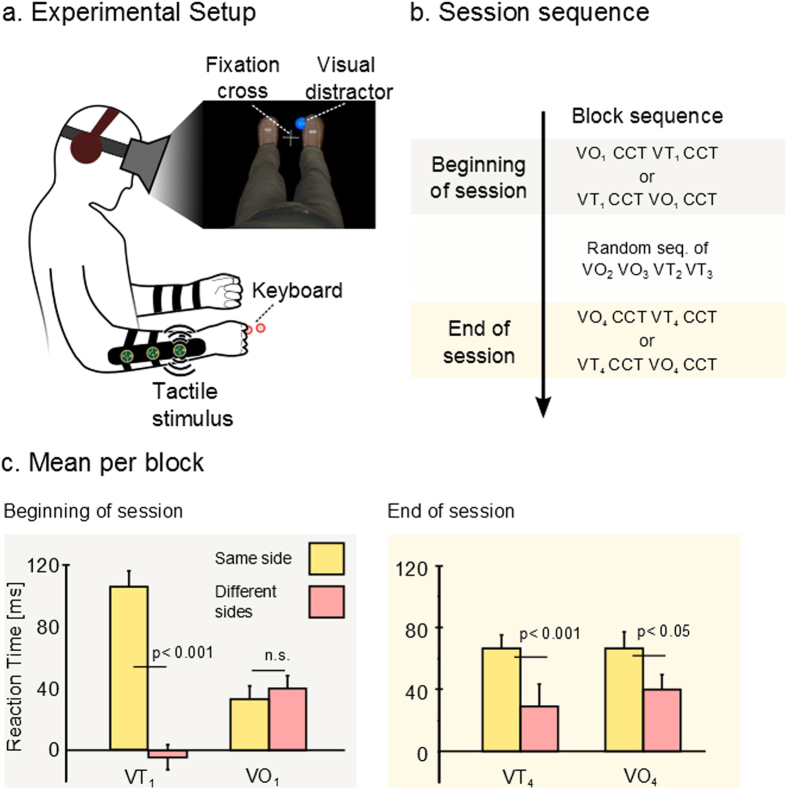
Crossmodal congruency task. (**a**) Experimental setup, subject wore the tactile shirt and the head mounted display. Vibrators were placed on patients’ forearms and visual distractors on the virtual avatar’s toe and heel. (**b**) A cross congruent task (CCT) block is run after a Vision only block (VO: 1 minute observation of the avatar walking) or a VT block (same as VO with tactile feedback on the stance). After a random sequence of 2 VO and 2 VT blocks, we ended the session with another sequence of CCT block following a VO or a VT block (**c**) Mean and standard error for the Cross Congruent Effect (CCE). Yellow bar shows average response time for trials where tactile stimulation and visual distractors were on the same arm and pink bar shows those where they were contralateral. P values for T-test are shown.

For all patients, at the beginning of the session, we ran a CCT block succeeding a first Vision Only block (VO_1_) and a CCT block succeeding a Vision + Tactile block (VT_1_) (Fig. 2a,b). CCE for the same side and CCE for different sides refer to the cases where the visual distractors and the vibration are on the same arm or on the contralateral arm. CCE(M_n_) refers to the Cross Congruent effect recorded right after the n^th^ block of a modality M. The mean reaction times (RTs), for all conditions over all patients, are shown in Table 1.

Mean CCE(VO_1_), calculated as the RT difference between trials where the tactile stimulation on the forearm and the visual stimulation on the avatar foot had incongruent elevations (e.g. vibration on the proximal vibrator and visual stimuli on the tip of the foot) and trials where they were congruent, was equal to 41 ± 8 ms (±SEM) when the vibrator and distractor were on a different side (incongruent: 828 ± 20[ms], congruent:787 ± 19 [ms]) and 33 ± 8 ms for the same side (incon: 755 ± 21 [ms], cong: 787 ± 18 [ms]).

We found that CCE(VT_1_) was −3 ± 8 ms for different sides (incon: 804 ± 21 [ms], cong: 807 ± 18 [ms]) versus 107 ± 8 ms for same side (incon: 855 ± 24 [ms], cong: 748 ± 17 [ms]). Three-way analysis of variance (ANOVA) with the factor congruency (congruent/incongruent), side (same side/different side) and modality (VT/VO) on RT, showed a significant effect of congruency (P < 0.01), and a significant effect of the interaction between side and congruency (P < 0.05). CCE was higher for same side than for different side delivery of distractor and tactile stimulation. We also found a three-way interaction between congruency, side and modality (P < 0.05). A post-hoc analysis with Bonferroni correction was run to determine the difference between CCE(VT_1_) and CCE(VO_1_). CCE was significantly higher for same side compared to different sides (P < 0.01) for VT modality. No difference was found for CCE(VO_1_) between the same and different sides (P > 0.5) (Fig. 2c). In other words, we found a significant CCE effect (used as the indirect measurement of the avatar leg assimilation) after the first VT block and no such effect was observed right after the first VO block.

Interestingly, when we repeated two more blocks of VO and VT (VO_2_, VO_3_ and VT_2_, VT_3_ randomly sequenced, Fig. 2b), and then ran CCE(VO_4_) and CCE(VT_4_), we observed different dynamics (see RTs in Table 1). The mean RT difference of CCE(VO_4_) was 34.4 ± 9.1 ms for different sides and 67.4 ± 7.3 ms for the same side; CCE(VT_4_) was 21.9 ± 9.0 ms for different sides and 69.5 ± 8.301 ms for the same side. Significant differences between the same side and different sides were found both in CCE(VT_4_) (P < 0.001) and CCE(VO_4_) (P < 0.05). Simply stated, there was a significant increase in RT during the CCE applied after a VO block at the end of the session; something that did not happen at the beginning of the session.

Altogether, these results suggest that patients extended their body representation to integrate the avatar legs after using the visuo-tactile stimulation.

### Simulation of Floor Texture

**Figure 3:**
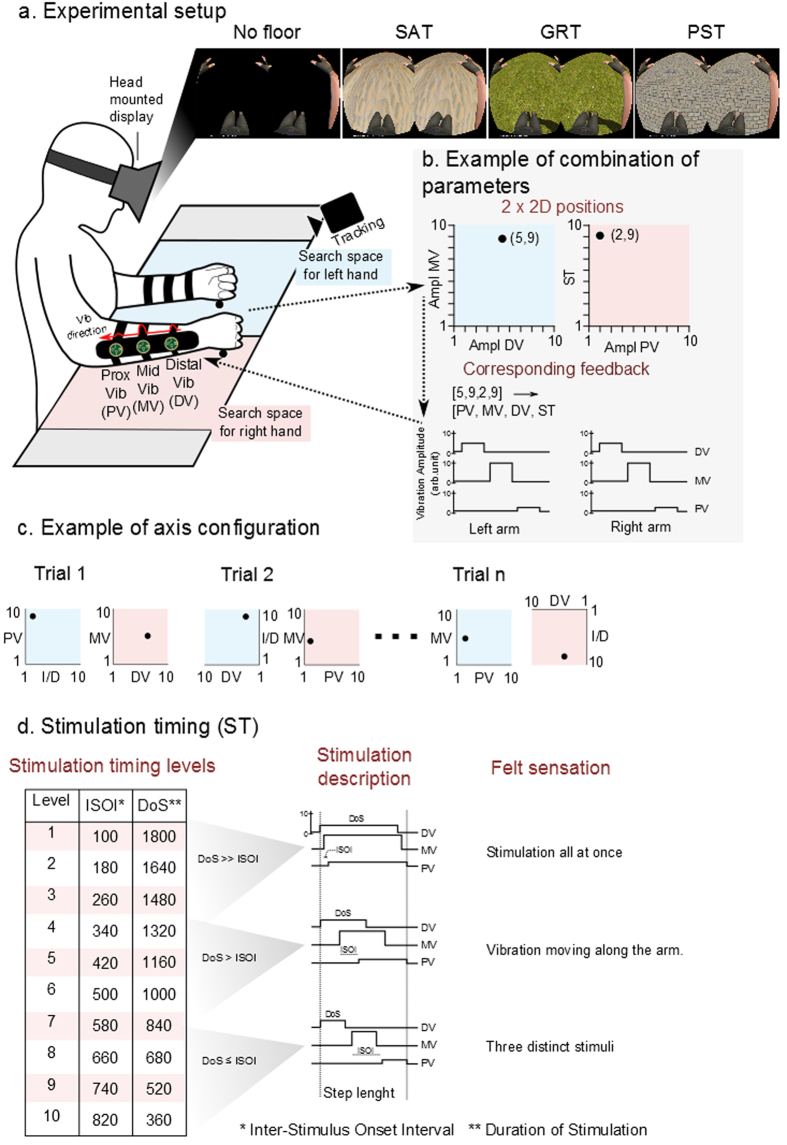
Floor texture simulation, setup. (**a**) Subjects wore the tactile shirt and the head mounted display; a 3D avatar was walking on either an empty floor or one of the three ground textures: Sand (SAT), Grass (GRT), and Paved Street (PST). (**b**) A tracking system detected the coordinates of the hand position and translated to a tactile modality defined by the amplitude of Proximal, Middle and Distal Vibrators (PV,MV,DV) and the Stimulation Timing (ST). (**c**) The axis of the four parameters describing the tactile feedback was randomized at each trial. Example of axis configuration and corresponding choices for SAT given by a subject. (**d**) Varying the Stimulation Timing (1 to 10) changes the Inter-Stimulus Onset Interval (ISOI) and Duration of Stimulation (DoS) parameters of the tactile feedback. It is possible to simulate the following sensation of touch: one single vibration on the whole forearm, a continuous feedback going from distal to proximal vibrator positions or three distinct vibrations on distal then middle and proximal forearm.

Patients explored and selected 120 times per session the set of tactile parameters that best represented for each of them the sensation of walking on three virtual ground textures labeled as: sand (SAT), grass (GRT) and paved street (PST) (exploratory phase). In other words, while seeing through the head mounted display, a black control surface or the images of three distinct ground surfaces (sand, grass, and pavement) upon which the avatar was walking, patients received a variety of tactile feedback patterns on their forearms. Four factors of the tactile feedback were varied to create a catalog of perceived tactile sensations: amplitude of the distal, middle and proximal vibrators (DV, MV and PV) and the stimulation timing (ST) (Fig. 3a–d). See Floor Texture Simulation Test section in Methods for a description of the procedure to explore the full catalog of sensations presented to the patient. This exploratory session lasted for 40–45 minutes. At the end of the session, a complete catalog correlating tactile stimulation patterns and ground surfaces was obtained for each patient.

**Figure 4:**
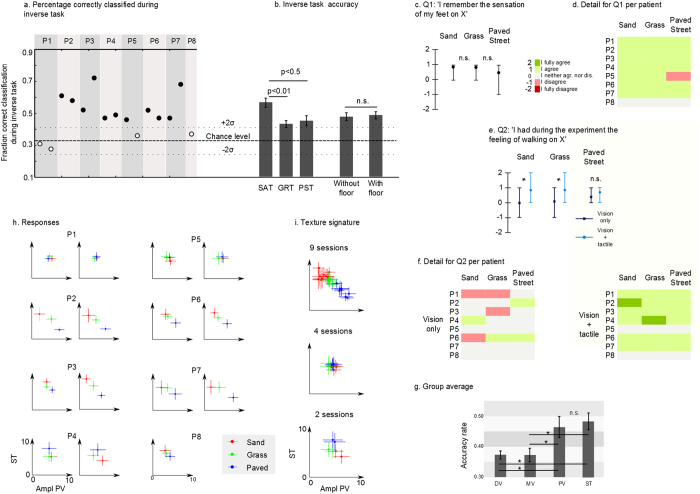
Floor texture simulation, results. (**a**) During the inverse task the parameters chosen during the exploratory phase were replayed in a random order; subject had to report the floor type relying on the tactile feedback only (chance level = 0.42). (**b**) Patients’ accuracy per floor type (multiple comparisons, N = 14) and for trials with or without floor. (**c**) Mean answer over all patients and responses to Q1 (asked prior to the experiment): ‘I remember the sensation of my feet on X’ (X: SAT,GRT or PST, ‘I strongly disagree’ −2 to ‘I strongly agree’ +2). (**d**) Detail of patients responses for Q1. (**e**) Subjects’ responses to question ‘I had during the experiment the sensation of walking on X’ after a V only session (patients observed the avatar walking) and a Vision + tactile session (tactile feedback on stance) (*for P < 0.05, Wilcoxon test). (**f**) Detail of patients’ answers to question Q2. (**g**) Principal tactile factors of the experiment, ground types are classified (using knn classification) using each one of the four factors. Mean classification accuracy over all sessions for four factors (multiple-comparison test, N = 14, *: P < 0.05). (**h**) Mean and standard deviation for principal factors: Amplitude of proximal Vibration (PV) and Stimulation Timing (ST) for all sessions. Color convention as follows: red for SAT, green for GRT and blue for PST. (**i**) Values from panel h are joined following structure similarities (e.g. sessions where SAT was on top left corner and PST on the lower right).

After a 20-minute break, we removed the floor from the virtual reality simulation and begin to replay the set of chosen tactile stimulation parameters in a random order, using the same haptic display applied to the skin of the patients’ forearm. Now we asked the same patients to report the floor type to which each set of tactile stimulation parameters corresponded (inverse task phase). Figure 4a shows that in 11 out of 15 sessions, the patients’ accuracy rate was significantly above chance (mean + 2σ = 0.42). In other words, most patients were able to correctly describe the ground texture originally associated with a particular set of tactile parameters used to stimulate their forearm skin. Overall, 6 out of 8 patients performed this task above chance: patients P2, P3, P4, P6, and P7 were significantly above chance in both of their sessions; patient P5 was significantly better than chance in one of two sessions. Details of the patients’ performance per floor type and session are shown in Fig. S4a. Mean accuracy was significantly higher than chance for all three surfaces (Fig. 4b). However, patients were better at finding SAT than GRT (multiple comparisons, Bonferroni correction, N = 14, P < 0.01) and PST (P < 0.05).

During the exploratory phase, in half of the trials, patients could see the avatar walking on a virtual ground that mimicked the one they were asked by the experimenter to identify (Fig. 3a), while in the other half of the trials no ground surface was visible under the avatar’s feet (no floor). No difference was found between trials in which the ground was shown and trials where the ground was absent from the virtual environment (T-test, P > 0.5) (Fig. 4b).

While all patients suffered a spinal cord lesion at least 3 years (and some as much as 10 years) prior to the onset of the experiment (Table S1), all patients except P8 reported still having vivid memories of the sensation produced by the interaction of their feet with sand and grass (Fig. 4c,d). For the sensation of walking on a paved street, all patients - except P5 and P8 – also reported such memories. After the experiment, six out of eight patients reported that they experienced again the vivid sensation of walking on the three chosen ground surfaces (Fig. 4e,f ‘Vision + Tactile’). P5 and P8 were the only patients that did not report this sensation. Patients’ responses were translated into numerical grades (Fig. 4e, −2 for ‘I fully disagree’ to 2 for ‘I fully agree’). The group average (N = 8) was positive for all three conditions: 0.88 for SAT and GRT, 0.75 for PST.

The patients’ responses were different if the same question was asked after a session where no tactile feedback was given (Fig. 4e,f Vision Only). In this control experiment, patients only observed the avatar walking, but did not receive tactile feedback. Patients’ answers were close to 0 for all three conditions when asked if they had the feeling of walking on the ground surfaces: paved street (0.25), grass (−0.13) and sand (−0.13).

Differences between “Vision Only” and “Vision + Tactile” were significant for SAT and GRT (Fig. 4e, Wilcoxon test, P < 0.05, N = 8), but not for the PST. At the end of “Vision Only” sessions, patients were also asked to report on the visual realism of the different ground surfaces. No significant difference was found among the three surfaces (P = 0.26, ANOVA). The ‘sensation of walking on X’ after a vision only session was not correlated with the patients’ report of the visual realism of the ground surface (Fig. S4b) (R^2^ < 0.01), suggesting that the vision of the avatar and the interaction of the avatar with the ground are more important than the visual realism of the ground itself.

Next, we analyzed the principal factors that patients relied on to differentiate the ground type (see Supplementary Methods). The stimulation timing (ST) was found to be the primary factor (Fig. 4g and S4c for details per patients) while the amplitude of the proximal vibrator (PV) came in second. Both were better than the middle (MD) and distal vibrator (DV, multiple comparisons, N = 14, P < 0.01). This suggests that patients relied on the tactile vibration level produced at the end of the avatar leg stance phase or the moment in which the foot pushed off the floor to move to a swing movement. Surprisingly, the patients seemed not to consider the vibration amplitude at the beginning of the stance (when the heel strikes the floor) as an important feature for discriminating the ground texture.

We used these two main factors (PV and ST) to characterize the way patients chose each ground type. Figure 4h shows the parameters’ mean and standard deviation for all three surfaces. Similarities can be seen in patients 3, 5, 6, 7 and 8 (shown in Fig. 4i). For SAT, these patients expect three distinct stimuli during stance with a light feedback when the foot pushes off the ground. On the contrary, for paved street PST they were looking for one long stimulation with a strong proximal vibration. GRT was somehow in between sand and paved street, with a middle range vibration amplitude for PV. The probability of these similarities being due to chance was found to be very low (P < 0.001, see Supplementary Methods).

Parameters chosen by the patients to describe the ground types were significantly different from each other for at least two textures in 10 out of 15 sessions (Table 2 and Supplementary Methods). The most common distinguishable textures were sand and paved street. No difference was found between trials where the floor was shown in the VR and those where it was not. Thus, the parameters selected by the patients to identify a given ground surface were not influenced by the presence of that ground in the virtual reality environment.

We also noticed that sessions where patients chose the same factors for all textures during the exploratory phase, namely Patient 1, Sessions 1 and 2; Patient 5, Session 2; and Patient 8, Session 1, correspond to the sessions where the subjects were not able to differentiate the floor textures during the inverse task. P1 was the patient with the lowest score in the sensory discrimination task (Fig. S3), and also the one with the lowest score in the Stance-DtP paradigm during the pseudo-proprioceptive test.

## Discussion

A novel solution to overcome sensory deficiency in the lower limbs for patients with Spinal Cord Injury (SCI) is proposed. In this study, missing haptic sensation from the lower limbs was replaced by rich tactile stimulation on the skin of SCI patients’ forearms. This feedback was integrated with an immersive virtual reality environment where a 3D human avatar was simulated. Three major effects of integration of the tactile feedback with the simulated 3D avatar were observed: (a) SCI patients could rely on this feedback to perceive the position of the virtual leg during locomotion; (b) patients incorporated the avatar legs as an extension of their body schema; and (c) patients experienced a realistic sensation of walking on different ground surfaces relying on the tactile feedback only.

Interestingly, patients were never instructed on what the tactile feedback displayed or represented. As totally naïve subjects, they acquired vivid tactile/proprioceptive sensations after exposure to 1 minute of synchronous visuo-tactile stimulation (patients observed the 3D human avatar walking and received the tactile stimulation on their forearm). This suggests that such tactile feedback became intuitive to the subjects rather quickly. As such, we propose that our haptic display paradigm was capable of inducing patients to experience a proprioceptive illusion that allowed them to deduce the position of the virtual avatar legs relying solely on the tactile feedback.

Human perception is multimodal^20^ and different sensory inputs are merged and weighted in a statistically optimal fashion in order to reduce the variance of the final estimate^21^. Each sensory input has proficiencies: vision can provide precise spatial encoding, audition is more likely to accurately convert temporal features, while the haptic sense can perceive both spatial and temporal information with high precision^21^. For this reason and because it is applied directly on the body, haptic feedback is believed to be the most intuitive augmented sensory feedback to describe limb movements, as it provides intuitive spatiotemporal cues directly on the limb thus reducing the level of abstraction required from the user to understand the movement^22^. Here, a haptic display proved to be capable of mapping the tactile/proprioceptive cues, describing bipedal walking, onto the skin of the patients’ forearm. Patients learned very quickly to link the haptic feedback to the avatar walking movement.

Multi-sensory training protocols have been reported to be more effective for learning^23^. Moreover, the benefits of a multi-sensory training phase are maintained further than when a single modality is used^24^. Similarly our protocol for integrating the tactile/proprioceptive feedback with an immersive virtual environment likely helped patients to internalize the relationship between a visually observed leg movement and the corresponding tactile stimulation in order to recall it once the visualization was removed. In this context, the emergence of this intuitive pseudo proprioception has an important implication for future use of brain controlled exoskeletons and other prosthetic devices, as the proprioceptive information is essential for controlling locomotion and other autonomous movements^25^,^26^.

Walking generates large amounts of parallel multisensory information streams that are used in the fine control of the gait cycle. Among these inputs, haptic information is essential as proven by our ability to walk with our eyes closed and inability to walk when tactile feedback from the legs is disrupted^27^. The effects of haptic perception during walking are complex as illustrated by the change in walking patterns resulting from a reduction of the tactile sensitivity in the foot sole^28^.

The importance of tactile feedback can also be appreciated when one examines the SCI patients’ perception of their own body. Sitting in a wheelchair after complete loss of sensory motor functions for several years changes the perception of one’s own body^29^. Experiments showed distortion of perceived body parts in SCI patients (they perceived a reduction in hip size^29^), as well as a decrease of body ownership of the legs and an increase in symptoms of depersonalization^30^. SCI patients were also found to consider their wheelchair as a ‘substitution’ of themselves or a part of their body^31^. These observations are not surprising as numerous experiments have shown that body representation in the human brain^15^,^32^ or primates^33^ modulates to incorporate additional limbs^34^, prosthetic limbs^35^,^36^, tools^37^, or virtual limbs^18^,^38^,^39^,^40^. The so called ‘rubber hand’ illusion^41^,^42^,^43^, which has been extensively studied, illustrates the plasticity of the brain representation of the body or body schema^38^,^41^.

In the present work, we attempted to answer the following questions: do SCI patients incorporate virtual legs? Is tactile feedback necessary for this effect to appear, or is the vision of an avatar resembling a human body enough? In response, we found that the incorporation effect was very quick and robust. The assimilation effect, recorded through well documented psychophysical measurement (CCE^15^,^18^), was observed after 1 minute of a congruent visuo-tactile stimulation. Additionally we observed that once the incorporation effect appeared, it was maintained for at least a few minutes (as shown by the maintenance of the effect after the last VO block).

Overall, our results are in concordance with previous findings on incorporation of upper limbs^15^,^42^,^44^ and lower limbs^19^ in healthy subjects or amputee patients^36^,^45^. We also extended these previous observations to SCI patients. Moreover, our findings create a bridge between limb incorporation and sensory remapping since our subjects integrated the avatar’s legs while receiving tactile feedback on their forearm.

Finally, reacquiring haptic feedback from the legs recreates the interaction with the external world, or in the case of walking, with the ground surface. Spinal cord injured (SCI) patients participating in this study found specific vibrotactile patterns that corresponded to the complex sensation of walking on sand, grass or paved street. The experimental paradigm allowed the patients to select among all possible discernable combinations of tactile parameters (10’000 combinations). The patients selected parameters which are easily clustered, showing that they had a clear idea of the sensation they were seeking. As a result, six out of eight patients performed at above chance identification rates.

One important question regarding these results is whether the patients associated a specific vibrotactile pattern to a given surface, or whether the vibrotactile pattern elicited a genuine sensation of walking on a specific floor. Several elements point towards the second hypothesis. The patients received no training, nor did they receive any feedback regarding their selections during the exploratory phase or the inverse task. Second, there was a break of at least 20 min between the exploratory phase and the inverse task; a delay larger than the one reported for haptic working memory (around 10 seconds)^46^. Finally, strong similarities in the selected parameters were found among patients, making it highly unlikely for their choices to be a mere repetition of a casual initial association.

Another interesting discovery of our experiment was the parameter that patients relied on the most to identify the ground surfaces. When the parameter corresponding to the structure of stimulation was somehow expected to be important (patients expected three separated stimulations when walking on a granular surface such as sand, a single impact when walking on a paved street and smooth stimulation for grass), it was surprising to see the second and only other important parameter was the sensation at the end of the stance. Patients considered the sensation of the foot pushing on the floor before starting a swing as a signature for describing the different ground types: strong feedback for PST, softer for GRT, lightest for SAT.

Our experiments highlight the importance of tactile feedback over vision for perception of floor texture. Presence or absence of the virtual floor in the virtual simulation did not influence patients’ choices of what they perceived as a certain ground type, although they all judged that the 3D simulation of the virtual grounds were realistic.

The subjective and conscious feeling, or the ‘kind of‘ sensation often referred to as *qualia*^47^, is an important aspect of our proposed approach to restore tactile perception. A more direct implementation of floor texture display could have been to sample material features from real-world surfaces and remap them through the tactile shirt^48^,^49^. We have opted for a different approach where naïve subjects search for their preferred sensation.

Lately, the nature of the subject’s perception during the use of sensory substitution devices has been questioned^50^: if someone uses an apparatus to perceive, through vibrators, a stream of visual data, can one conclude that the user is ‘seeing with the skin’? While our work does not pretend to permanently solve this debate, we believe that our experimental paradigm avoided some of the issues that make interpretation of sensory substitution devices tedious. Our patients did not learn to discriminate among different stimuli; they reported the one they considered closest to their perception of walking on different surfaces. As a result, the majority of patients described the sensations as realistic and similar to the one of walking on the corresponding floor type. The patients that did not find the sensation realistic were also the ones that reported not remembering walking on some of the floor types, suggesting that the sensation found during the experiment was linked to their personal experience of walking on these floors prior to the SCI. One patient once spontaneously reported ‘I was walking in a happy mood, because I was walking on the beach’.

Altogether our experiments show important emergent positive effects when chronic SCI patients take advantage of our tactile shirt and highlight the importance of this feedback for BMIs and neuroprosthetics to be clinically relevant. In addition to the assistive benefits during locomotion, allowing patients to rely less on visual feedback, by relying on tactile feedback, patients can look forward instead of looking down at their feet to know where they are in their walking cycle. Continuous use of the haptic interface seems to change the patients’ body schema by altering their cortical representation of lower limbs through the process of incorporation of the virtual avatar legs.

Interaction with our haptic interface also induced in the SCI patients a more vivid sensation of walking and the return of interactions with part of their peri-personal world that had been lost many years prior, i.e. the space immediately under their feet. To some degree, by reacquiring the ability to experience contact with the ground, and to perceive different types of ground surfaces, our haptic interface provided the patients with a much richer lower limb “phantom” sensation. Such an enriched illusion likely contributed to making these patients much more amenable to the idea of walking with the assistance of a custom-designed robotic exoskeleton^7^, since their walking experience with this orthosis generated a much more realistic sense of bipedal locomotion that they had experienced since their SCI.

## Methods

This study’s protocol was approved by the ethics committee of AACD (Associação de Assistência à Criança Deficiente, São Paulo, Brazil) and carried out in accordance with its guidelines. All participants provided written informed consent before enrolling in the study.

All our patients were initially evaluated using the American Spinal Injury Association (ASIA) Impairment Scale (modified from the Frankel classification) to quantify the severity level of their spinal cord injury. This scale grades SCI from ASIA A, for a complete lesion with no sensory or motor function below the neurological level of the injury, to E, for normal sensory and motor functioning^1^. Seven ASIA A patients and one ASIA B patient, all in the chronic phase of the SCI (at least one year after the SCI), were selected as subjects for all experiments reported here (see Table S1 for patients’ demography). All our patients had lesions below or equal to the thoracic dermatome T4. Accordingly, they all exhibited normal sensory motor functioning in the upper limbs.

Three different psychophysical experiments were performed in the course of 6 months: a pseudo-proprioception test, a cross congruent task and a task involving the simulation of floor textures. Globally, these experiments were designed to: (a) provide the patients with an immersive visuo-tactile experience of walking; and (b) assess the impact of an augmented somatosensory feedback on the patients’ perception of their own body.

During all experiments patients were seated in their wheelchair while wearing a tactile shirt (see Tactile Shirt description). In all the experiments, patients also wore a head mounted display on which a 3D human avatar was projected. The avatar could stand and walk and, as it performed these movements, tactile feedback, reproducing the touch of the avatar feet on the ground, was delivered on the skin of the patients’ forearm through the employment of a haptic display (e.g. the tactile shirt: see Integration of the virtual body avatars with the tactile shirt).

In the pseudo-proprioception task, we delivered four distinct tactile feedback paradigms emulating different features of the avatar’s walk and determined which paradigm was the most intuitive for the patients.

The Cross Congruent task (CCT)^15^,^37^ was used to explore the brain representation boundaries of the body schema in our patients after they used the tactile shirt. Finally, by simulating floor textures, we investigated whether spatiotemporal changes in the patterns of forearm tactile stimulation could give patients the perception of walking on different types of surfaces, like grass, sand or a paved street. Because there is no *a priori* answer for how to stimulate someone’s forearm in order to render the complex sensation of walking on different floor surfaces, we proposed a novel approach to search the best vibro-tactile parameters to render the floor types without making any assumption on the user’s perception.

### Virtual Reality Environment and Setup

Three virtual human avatars (one woman and two men) were modified based on free online stock models from Maximo (Maximo Inc. 2015). The virtual avatars were animated to walk and stop; animation blending was accomplished using MotionBuilder (Autodesk Inc. 2015). Custom written C++ code controlled the triggering of avatar movements, the type of surface where the avatar walked and calculated the interaction of the avatar with the surface. In addition, we integrated the Oculus rift (Oculus VR) head mounted display with MotionBuilder using a technique called OpenGl intercept^51^.

### Tactile Shirt as a Haptic Display

To deliver artificial tactile and proprioceptive feedback signals, originating from the movements of the human 3D avatar, we created a haptic display embedded in the long sleeves of a shirt. This haptic display was named “tactile shirt” and employed eccentric mass (ERM) vibrators to deliver somatosensory feedback to the skin of the patient’s forearm. The ERM vibrator consisted of a DC motor rotating an eccentric mass at different angular velocities, allowing the generations of various amplitudes and frequencies of vibration. ERM frequency and amplitude were coupled, and the maximum stimulation amplitude was reached at about 150–250 Hz, which corresponds to the peak response frequency of Pacinian Corpuscles (a type of rapidly adapting mechanoreceptor which is sensitive to mechanical transients and rapid vibrations in the range of (~40–400 Hz)) in the human hairy skin^52^. Our tactile shirt used three coin-shaped, 2 cm diameter, ERM vibrators to deliver sensory feedback, reproducing ipsilateral lower limb tactile or proprioceptive signals, to each of the patients’ forearms (Fig. 1a)

The three vibrators were placed 6 cm apart from each other along patients’ forearms, following the longitudinal axis of the ulna bone (Fig. 1a).

Since all our patients had thoracic lesions (T4-T11), in theory none of them should exhibit sensory deficits in the forearms. Preliminary tests showed that 6 out of 8 patients could discriminate vibrator position with 50 ms-long vibrations on their forearms (Fig. S3). We decided to move the shirt to the ventral part of the forearm for Patient P6, who had difficulties feeling stimuli on the ulna. Patient P1 was found to have difficulties discriminating vibration pulses under 70 ms, so longer pulse trains were employed with this patient.

### Integration of the Virtual Body Avatars with the Tactile Shirt

During the experiments, patients sat in a wheelchair, wearing an Oculus Rift head-mounted display (HMD) (Oculus, VR) in which virtual legs were projected, mimicking the position and orientation of the patients’ own bodies (Fig. 1a). Patients also wore headphones playing white noise to avoid biases due to noise from the vibrators. Prior to the first experiment, we ran two tests with the head-mounted display to evaluate if all patients could perceive correctly the 3D Virtual Reality (VR). We also evaluated whether any of them experienced any sign of motion sickness (Fig. S5). One subject experienced strong motion sickness during the second test while quickly moving the head. Thus, to have a single setup suited for all patients, we kept a fixed camera point of view for all patients during all the experiments.

### Pseudo Proprioception Test

We tested two different feedback modalities related to different stages of the avatar body locomotion and assessed which one was more intuitive for patients to perceive the position of the avatar leg when relying on tactile feedback only: (a) feedback given during the stance phase of the virtual avatar legs; (b) feedback given during the swing phase; as well as two directions of tactile stimulation on the forearm: proximal to distal (PtD) or distal to proximal (DtP).

The four combinations given by the two modalities x two directions were tested in separate experiments (randomized order), with each one divided in four blocks (Fig. S2a).

The first block lasted 30 seconds during which the patients were looking at the virtual avatar legs through the HMD. At this stage, the avatar walked at medium speed of 66 steps per minute; patients did not receive any tactile feedback on their forearms. They were asked to look at the avatar legs and imagine that they were their own.

The second block lasted 1 minute and included the delivery of tactile stimulation on the forearm skin surface, in synchrony with the avatar walk and according to one of the four tactile conditions. For the third block, the HMD was turned off while the avatar continued walking at the same speed and tactile feedback was delivered accordingly. Patients were asked to rely on the tactile stimulation while imagining their own legs moving. This block lasted 30 seconds. After the third block, the avatar stopped walking, and after a 20 second break the last block started. Here, subjects were instructed to match the upcoming avatar walk, provided through tactile feedback, with a corresponding movement of their arms. The avatar resumed walking, first at 66 steps per minute. The walking speed was randomly reduced to 50 steps per minute or increased to 100 steps per minute without the user’s knowledge and without specific time patterns (Fig. S2a). A camera filmed the arms of the patient during this exercise and compared these to the joint positions of the avatar legs.

A score, ranging from 0 to 3, was given to evaluate a patient’s performance for each speed. A 0 score was given if the patient’s arm movements were not synchronized with the avatar walk at any phase for a certain speed. A score of 1 was given if a patient managed to follow the walk at constant speed, but did not manage to follow the walk when the avatar’s walking speed changed (for example from medium to high speed). A score of 2 or 3 was given if the patient managed to follow the avatar walk synchronously during constant and changing speeds. Scores differed when patients were synchronous with the avatar walk but exhibited a contralateral inversion (for example showing the stance with the left arm during right leg stance and vice versa, score 2), and when patients were synchronous and used the correct arm to show the correct leg (score 3).

We ranked the four tactile paradigms Pi based on the following scoring:

Score(Pi) = ∑ patients who had their best score with Pi – Σ patients who had their worst score with Pi. At the end of each test, patients answered a questionnaire (Fig. S2b) and restarted a new test with a different tactile paradigm.

### Cross Congruent Task (CCT) test

To measure whether the virtual avatar leg was “incorporated” by the patients, we ran an adapted version of the cross congruent task (CCT)^15^,^18^,^53^. This task is based on the observation that human subjects are slower in detecting a tactile stimulus on the index finger if a visual distractor appears close to the thumb (and vice versa)^54^. This effect, named crossmodal interference, is stronger if the distractor is placed on the same hand as the tactile stimulation than when the visual distractor and tactile stimulation are contralateral. Similarly after active use of a tool, the multimodal interaction between visual stimuli on the tip of the tool and tactile feedback on the user’s hand changes^55^: visual distractors on the tip of the tool led to an increase in response time (RT) when the vibration and the distractor were incongruent compared to the congruent case. These findings were interpreted as revealing the incorporation of the tool as an extension of the subject’s arm, due to the projection of cortical visual receptive fields to the distal edge of the tool.

Patients wore the tactile shirt while observing the 3D avatar through the head-mounted display (Fig. 2a). A trial started with a visual fixation cross placed between the avatar’s feet for 1000 ms. Next, a 50 ms vibration was randomly triggered in one of four locations: proximal or distal location of the left or right forearms. Light distractors (3 cm radius 3D blue spheres) appeared 100 ms before the mechanical vibration in one of the four locations: left/right toe/heel of the avatar leg (100 ms offset was found to maximize the crossmodal interaction^56^). Patients were asked to indicate, using two keys on a keyboard, whether a vibration was delivered on the front part (distal) or in the back (proximal) of the forearm while ignoring the visual distractor.

Each session started with 5 minutes of training. The experiment started only if patients had >85% accuracy in detecting the correct position of vibration during this training. The experience consisted of 5 minute long CCT blocks run immediately after subjects experienced 1 minute of observation of the avatar walking (Visual Only, VO), either with simultaneous tactile feedback moving from wrist to elbow of the patient’s forearm or when the ipsilateral avatar foot was in contact with the floor (Vision + Tactile feedback, VT) (Fig. 2b).

For each CCT block we tested all 16 configurations of tactile feedback (four positions) and visual distractor (four positions). On each block CCT we repeated each configuration four times. We repeated the same experiment for all patients.

Response time (RT) was measured and trials with RT longer than 1800 ms or faster than 200 ms were discarded as well as trials with RT beyond the range of mean ± 3 × std per (8.5% of the overall trials of all sessions).

### Floor Texture Simulation Test

We investigated the set of tactile parameters that could be used with our tactile display in order to induce the illusion of walking on three different ground surfaces: sand (SAT), grass (GRT), and paved street (PST). We then compared the parameters obtained for all patients.

Patients were seated in their wheelchairs wearing the tactile shirt (see Fig. 3a) and a head mounted display. A 3D human avatar was shown in first person view. The avatar and the virtual environment were rendered using MotionBuilder (Autodesk) software. The avatar’s walking was based on motion capture of a healthy subject walking at 45 steps per minute.

We presented a catalog of textures to our subjects. Patients were asked to choose those that best represented the sensation of walking on SAT, GTR, and PST. The catalog was created by varying four parameters describing the tactile stimulation: the amplitude of the distal vibrator (DV), the amplitude of the middle vibrator (MD), the amplitude of the proximal vibrator (PV) and the stimulation timing (ST) (Fig. 3a,b). Each one of these four factors had 10 possible levels. The number of possible combinations was thus 10^4^ = 10’000. Vibrator amplitude 1 represented the lowest perceived sensation, and 10 the strongest before sensory saturation (both found empirically).

The stimulation timing was defined by two factors: Duration of the Stimulus (DoS) and the Inter-Stimuli Onset Interval (ISOI). ISOI represents the time between onset of one vibrator and the next. The DoS was chosen to satisfy the following relation: DoS = Stance Duration – 2xISOI; where Stance Duration was fixed to 2000 ms throughout the entire session. ISOI varied between 100 ms and 820 ms. An ST level 1 referred to the shortest ISOI and longest DoS (Fig. 3d) and corresponded to three long vibrations delivered almost simultaneously. For an ST of level 10, the onset asynchrony was longer than the stimulation duration, resulting in three short and distinct vibrations (not overlapping). Between these two extremes some levels of ST produced a continuous moving touch illusion known in haptics as apparent movement^16^.

Each experiment started with patients seated in front of a table wearing the head-mounted display to observe the 3D avatar and with their arms placed on a table (Fig. 3a). A thick tape was used to delineate two square areas of 50 × 50 cm^2^ on the table. Patients were asked to keep the left hand inside the left square and right hand in the right square. A tracking system recorded the position of both hands in the two referential areas defined by the two squares. As patients moved their hands over the two square areas, they received a particular pattern of tactile stimulation, defined by the four factors described above (Fig. 3b). More specifically, planar, Cartesian coordinates of the left and right hand were mapped onto four tactile parameters. Hence, each spatial position inside the two squares was assigned with a particular pattern of tactile stimulation to the patient’s forearms. Axes were randomized at each trial.

The session started with a 15-minute training phase. The experimenter asked the patient to find the tactile feedback that represented best for her/him walking on sand or grass or paved street. Patients freely explored the 2D spaces defined by the two squares. As they explored this virtual “tactile space”, patients were asked to observe the avatar walking on a black floor. Tactile feedback was delivered via the tactile display during the stance phases of the avatar on the ipsilateral arm.

Next, an exploratory phase started where the same procedure was repeated 40 times per surface (a total of 120 trials), in a randomized order. To avoid patients learning the position of a certain texture on the table, the four axes on the table were randomized for every trial (four possible axis configurations for left side, four for right side and all possible permutations of the four parameters = 4 × 4 × 4! = 384 possible configurations, Fig. 3c). Here, instead of the experimenter announcing the floor type, a cube appeared for 5 seconds in the Virtual environment (VE) at the beginning of each trial: red for SAT, green for GRT and blue for PST. For half of the trials (randomized order), immediately after the cube disappeared from view, the corresponding floor – sand, grass, or paved street - was displayed in the VE (Fig. 3a). For the other half of trials, the floor stayed black. Patients had 2 minutes to explore the table (and thus the catalog of textures) and confirm that the correct sensation was identified by raising their right hand by 10 cm. The task was the same in the visual presence or absence of the corresponding floor in the VE. After confirmation, the avatar was stopped. Following a 2–5 second inter-trial time, a new colored cube appeared and a new trial started. The trials were divided as follows: first block of 36 trials, a break of 5 minutes; a second block of 36 trials, a break of 45 minutes; and a third block of 48 trials. Inside a block there were always the same number of trials with and without floor and the same distribution of surface types.

After the exploring phase was concluded and patients had a 20 minute break, the next step of the experiment started. The experimenter played back in the haptic display applied to the patients’ forearms the 120 textures chosen during the searching phase. Patients had to say which floor type the avatar was walking on or say ‘I don’t know’. The patient held their arm on the table and, using the head-mounted display, observed the avatar walking on an empty black floor. This phase was named the inverse task. At the end of the first session a questionnaire was administered:

Q1) During the experiment I had the impression of walking on SAT, GRT, PST.

Q2) I remember the sensation of my feet on SAT,GRT, PST.

Two months later we ran a control experiment with all patients. The patient observed the avatar walking on the three different floors for 15 minutes. No tactile feedback was used. The session was followed by a questionnaire containing question Q1 and the following question:

Q3) The floor of type SAT/GRT/PST I saw in the head-mounted display was visually realistic.

## Additional Information

**How to cite this article**: Shokur, S. *et al*. Assimilation of virtual legs and perception of floor texture by complete paraplegic patients receiving artificial tactile feedback. *Sci. Rep.* **6**, 32293; doi: 10.1038/srep32293 (2016).

## Supplementary Material

Supplementary Information

## Figures and Tables

**Table 1:** Mean Response Cross Congruent Effect over all patients.

			Congruent	Incongruent	Mean CCE
Beginning of the session	VO1	Diff side	787.1 ± 18.6	828.1 ± 19.9	41.0 ± 8.0
		Same side	755.4 ± 20.8	789.3 ± 19.7	33.9 ± 8.3
	VT1	Diff side	807.8 ± 18.4	804.4 ± 20.6	−3.4 ± 8.1
		Same side	748.1 ± 17.2	855.8 ± 24.4	107.6 ± 8.4
End of the session	VO4	Diff side	820.7 ± 22.2	855.1 ± 22.0	34.4 ± 9.1
		Same side	762.4 ± 15.5	829.7 ± 21.2	67.4 ± 7.3
	VT4	Diff side	815.9 ± 24.5	837.8 ± 21.9	21.9 ± 9.0
		Same side	792.0 ± 19.8	861.5 ± 23.4	69.5 ± 8.3

Mean ± standard error of reaction time (in ms) over all eight patients for blocks succeeding a Vision Only (VO) block or blocks succeeding a Vision + Tactile (VT) block. Mean CCE corresponds to the mean RT for congruent – mean RT for incongruent visuo-tactile simulation is show in the right- most columns.

**Table 2:** ANOVA on Euclidian distance between chosen parameters for floor textures.

Patient	Session	SAT vs. GRT	SAT vs. PST	GRT vs. PST
1	1			
1	2			
2	1		***	
2	2		***	
3	1			***
3	2	***	***	***
4	1			
4	2	*		
5	1			
5	2			
6	1		***	
6	2	*	***	
7	1		***	
7	2	**	***	***
8	1			*

For each pair of texture i and j we compared the distribution of the Euclidian distances of all combinations of trials for both textures to the distribution of Euclidian distance of trials of type i and trials of type j. We reported stars in the cell only when the distance between the textures was significantly bigger than the distance between trials for each texture taken individually (*for P < 0.05, **for P < 0.01, ***for P < 0.001).
