# Supplementary material for: Assimilation of virtual legs and perception of floor texture by complete paraplegic patients receiving artificial tactile feedback
Source: Sci Rep. 2016 Sep 19;6:32293. doi: 10.1038/srep32293 (PMC5027552; doi:10.1038/srep32293)
Supplement: Supplementary Information [file srep32293-s1.pdf]

SUPPLEMENTARY MATERIAL

ASSIMILATION OF VIRTUAL LEGS AND PERCEPTION OF FLOOR TEXTURE BY COMPLETE PARAPLEGIC PATIENTS RECEIVING ARTIFICIAL TACTILE FEEDBACK

Solaiman Shokur[1], Simone Gallo[2], Renan C. Moioli[3,4], Ana Rita C. Donati[1,5], Edgard Morya[3,4], Hannes Bleuler[2], Miguel A.L. Nicolelis[1,3,4,6,7,8,9,*]

[1] Neurorehabilitation Laboratory, Associação Alberto Santos Dumont para Apoio à Pesquisa (AASDAP), Sao Paulo, Brazil.

[2] STI IMT, Ecole Polytechnique Federal de Lausanne, Lausanne, Switzerland.

[3] Edmond and Lily Safra International Institute of Neuroscience, Santos Dumont Institute, Macaiba, Brazil.

[4] Alberto Santos Dumont Education and Research Institute, Sao Paulo, Brazil.

[5] Associação de Assistência à Criança Deficiente (AACD), São Paulo, Brazil.

[6] Department of Neurobiology, Duke University, Durham, NC, USA.

[7] Department of Biomedical Engineering, Duke University, Durham, NC, USA.

[8] Department of Psychology and Neuroscience, Duke University, Durham, NC, USA.

[9] Center for Neuroengineering, Duke University, Durham, NC, USA.

Supplementary Methods

Apparent Movement

The apparent movement of a tactile stimulus is perceived when a number of stimulators are aligned on the user's skin and then activated sequentially. In our setup, the stimulators (i.e. the ERM vibrators of the long sleeves of the tactile shirt) were aligned and spaced on the user's forearm skin surface and activated sequentially according to their position.

Experiments started by optimizing the parameters of tactile feedback (in terms of duration of stimulation per vibrator and time interval between stimulation of two adjacent vibrators) to induce the apparent movement illusion in all our patients.

We tested which combinations of Duration of Stimulation (DoS) and inter-Stimuli Onset Interval (ISOI) would induce the most salient apparent movement sensation by varying the overall duration of skin stimulation in all eight patients. While wearing the tactile shirt, subjects were presented with apparent movement durations (DoApM) ranging from 400 ms to 1800 ms. Using three vibrators, the DoApM was equal to 2xISOI + DoS. Following this linear relation, all possible combinations of ISOI and DoS were derived for each presented DoApM. During each trial, the subjects were able to modify the stimulation by pressing a key that incremented the value of DoS by 50 ms (or 25 ms for ISOI). The subjects reported orally which stimulation yielded the strongest impression of continuous movement. DoApM values of 400, 600, 1000, 1400 and 1800 ms were tested for a sequence of vibration triggered from both Proximal to Distal (PtD) and Distal to Proximal (DtP).

For all eight subjects, we computed a linear regression on the DoS values and

observed a linear relation between DoApM (going from 400 to 1800 [ms]) and DoS (or ISOI) in terms of inducing the apparent movement sensation (coefficient of determination $R^2 > 0.95$ for all subjects). Two analyses of variance (ANOVA) were performed with the slope and the offset of the linear fit as dependent variables for each ANOVA to analyze the effect of direction of stimulation (PtD versus DtP). The independent variable was the direction of the apparent movement (2 levels: PtD and DtP). No effect was found for either the slope or the offset (both $p > 0.05$). Means and standard errors of DoS over all patients are shown for the different DoApM (Fig. S6A-B). The low standard errors suggest that the mean can be used as a good estimator of the DoS (therefore ISOI) for any stimulation duration, for all patients. A linear regression was then computed on the mean DoS ($R^2 > 0.95$). Thus, we found that the same linear regression can be used as an estimator of DoS (and ISOI) to generate realistic apparent movements for any duration of stimulation in the studied range for all subjects.

Principal parameters to define the floor texture

In the simulation of floor texture experiment, we searched for the parameters upon which patients relied the most to choose each ground texture. For this we ran a k nearest neighbor classification algorithm considering each one of the four tactile parameters (amplitude of proximal, middle and distal vibrator and stimulation timing). We considered all trials over all sessions at once (N = 120 x 15 = 1800). Data was bootstrapped 100 times in training and testing set (ratio 5 to 1). Mean per parameter and session is shown in Figure S7A.

Responses similarity probability

We calculated the probability of serving similarities among patients' responses clusters. Considering the following partition of the PV/ST referential: top left and right area, lower left and right area and the central area, there are $5^3 = 125$ possible configurations for placement of the centroid of the three textures. Considering all configurations had the same probability p (1/125) to be chosen, the probability P of having at least m times a configuration over n trials can be calculated using the probability mass function of the binomial distribution B(n,p):

$$f(k,n,p) = \binom{n}{k} * p^k * (1-p)^{n-k}$$

$$P = \sum_{k=m}^{n} f(k,n,p)$$

.

In the experiment we observed that in 9 out of 15 sessions, subjects chose the same configuration. The probability of this sequence is thus:

$$P = \sum_{k=9}^{15} \binom{15}{k} * \left(\frac{1}{125}\right)^k * \left(\frac{124}{125}\right)^{15-k} \cong 0$$

Parameter separability for floor texture simulation test

To analyze how patients chose the factors that defined a given virtual ground surface in the presence or absence of a virtual ground, we calculated for each subject the average difference of chosen factors between the repetitions of trials for each condition. More specifically, we derived the vector Tij representing the distribution of Euclidian distances between Ti and Tj, where Ti,j are vectors containing the 40 sets of four tactile parameters obtained in the 40 trials where surfaces ti,j were presented. For example, the distribution T_{12} of

distances between the ground types t_1 and t_2 is given by the Euclidian distance of the four factors between all combinations of trials m of T_1 with surface type t_1 and all trials n of T_2 with surface type t_2. Notice that the maximum distance is given by the diagonal of the 4D hypercube with edges of size 10 (there are 10 levels per factor), thus $\sqrt{4} \times 10 = 20$. The number of repetitions per floor type was 40, so that any vector T_{ij} had $40 \times 40 = 1600$ elements. We also calculated the distribution of distances within a surface type, i.e. Tii or Tjj. T_{11} is the distribution of distances between all combinations of two trials $m, n, m \neq n$ for surface t_1. T_{11} has $1600 - 40 = 1560$ elements. To analyze separability between surface t_1 and t_2 we ran a multiple comparison test between T_{12}, T_{11} and T_{22}. We also checked if the mean distance between surfaces 1 and 2 was larger than the mean distance between elements in surface 1 and those of surface 2.

Tactile Shirt hardware

The haptic display was based on a latching system featuring circular printed circuit boards (PCBs), the actuation and bridge boards, snapped together using metal snap buttons. Each ERM vibrator was mounted on the actuation board, on which four female metal snap buttons were soldered. The actuation board also contained the driving electronics for the actuators (FAH4830, Fairchild Semiconductor, USA). The bridge boards were attached on the desired locations of the shirt's long sleeves. They featured four male snap buttons corresponding to the ones on the actuation boards. By aligning the snaps on the two boards and pressing them together, the actuation board was latched to the long sleeves of the tactile shirt.

Both the actuation board units and the bridge boards had a diameter of

18 mm, allowing sufficient resolution for actuation placement. An Arduino Board, and the LilyPad SimpleSnap, featuring a 32-bits Atmel ARM processor, were employed as the shirt's central control unit, which powered and controlled all the actuators through thin and flexible 4-pin flat cables connected to each bridge board. The various sensory feedback paradigms used in this study were coded directly in this central unit. The tactile shirt was autonomous both in terms of power and control. Moreover, the shirt could be washed easily, once the actuation boards were unsnapped, as the bridge board contained no water-sensitive electronics.

The tactile shirt provided tactile/proprioceptive feedback to patients both during training in virtual reality and training with a mechanical prosthetic device. In the first case, the walking phase was generated by the virtual reality controller itself. Communication with the tactile shirt was performed through RS232 serial communication. In the second case, force and distance sensors where interfaced with the tactile shirt to detect walking phases (stance and swing) of the mechanical prosthetic device. Corresponding tactile feedback was displayed through the tactile shirt.

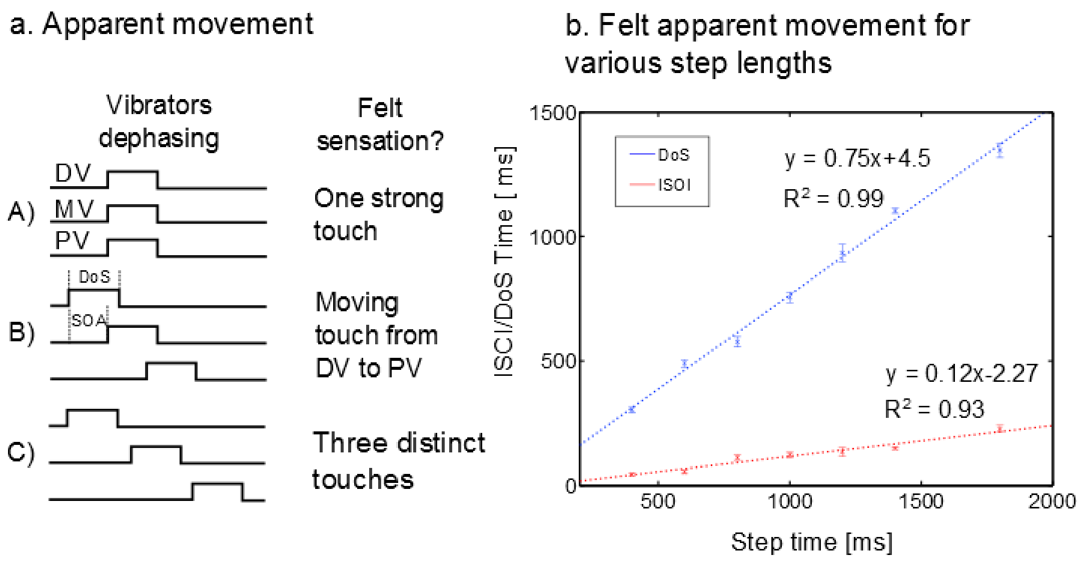

Fig. S1. Apparent movement. (a) Description of temporal tactile parameter to perceive a continuous (apparent movement) when being stimulated by three equidistant vibrators: if all three tactile stimulations (DV, MV, PV describe distal, medium and proximal vibrators) are simultaneous the subject has the feeling of one strong touch; in the other extreme, if the three vibration are well separated, the subject will perceive three distinct touches. In these two cases there are values for the Duration of Stimulation (DoS and the Inter Stimuli Onset Interval (ISOI) that will give the sensation one continuous touch going from DV to PV. (**b**) Different combinations for DoS and ISOI values were varied and patients were asked to report if they perceived a continuous touch. Mean and std of the DoS and ISOI corresponding to the stimulations classified as 'apparent movement' by the patients (mean over all patients) is shown for seven different step lengths between 400 and 1800 ms. Linear regression and coefficient of determination R^2 are shown.

a

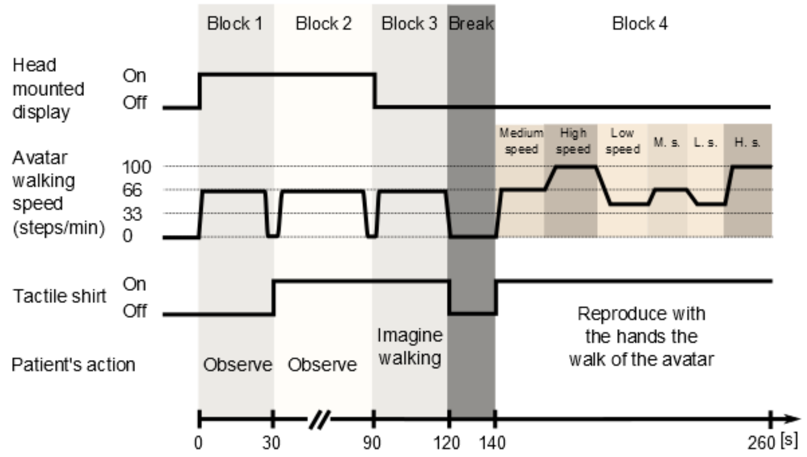

b

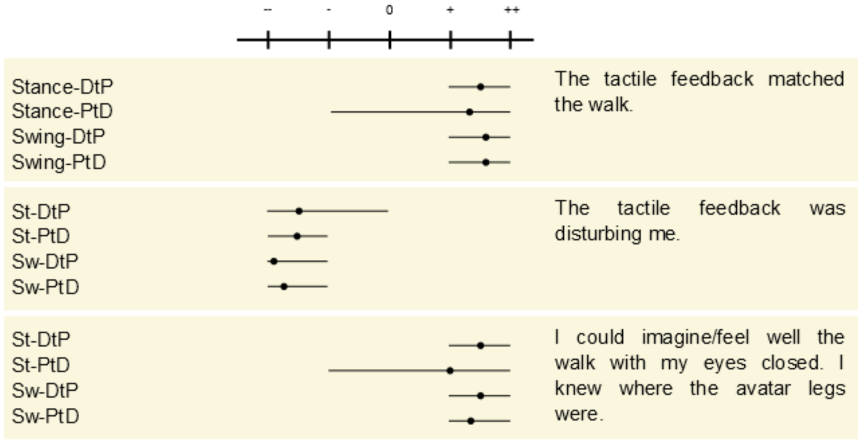

Fig. S2. (**a**) Pseudo-proprioception test blocks. On first and second blocks the head mounted displayer is on and patient can see a 3D human avatar. The avatar walks during block 1-3 and stops in between blocks and during the pause block. Then in 4th block avatar starts a medium speed; experimenter randomly increases or decreases the speed of the avatar's walk. The patient has to reproduce with their arm the perceived position of the avatar legs relying on the tactile feedback only. The tactile shirt displays feedback corresponding to the avatar walk on the patient forearm skin on all blocks except the first. Timeline in [s] is shown. (**b**) Questionnaire results for finding most intuitive

tactile paradigm. Mean and responses range for all patients. Questionnaire was run immediately after each experiment. Patients rated from -- ('I fully degree') to ++ ('I fully agree').

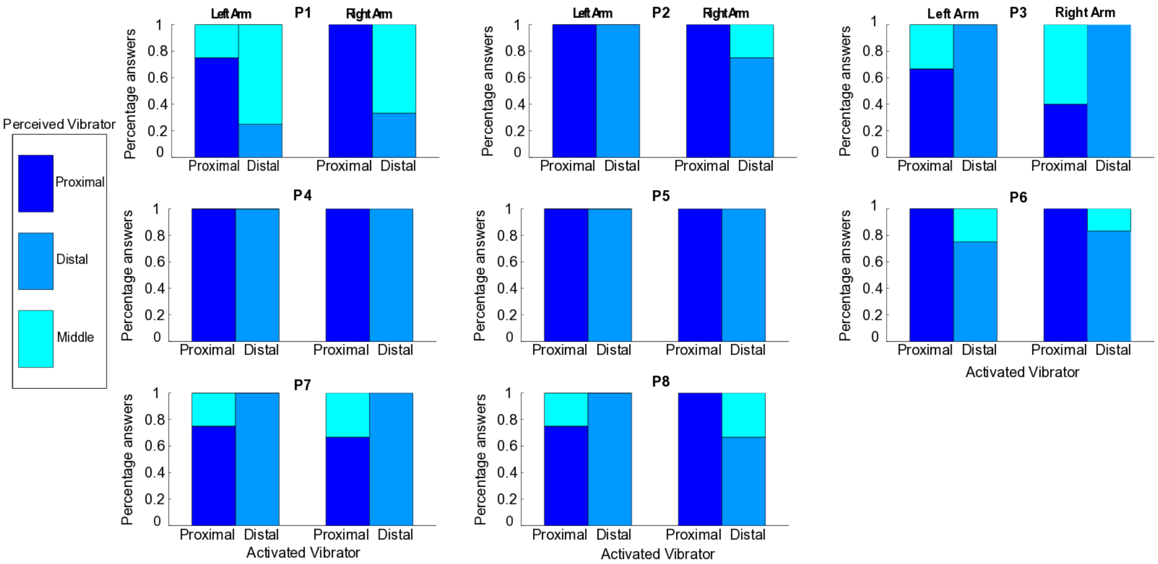

Fig. S3. ***Tactile characterization test*****: Percentage of perceived vibrators in stimulus localization test.** A subjective test was performed to assess the patients' ability to correctly locate a vibrotactile stimulus. The three vibrators were placed 6 cm apart on the forearm of the patients close to the Ulna bones. Their arms were resting on a table and a veil placed over the arms was used to eliminate visual cues. Only the proximal and the distal vibrators on both arms were activated but the patients were told that any of the six vibrators could be activated. Prior to the experiment all the vibrators were activated sequentially to check whether the patients were able to detect each vibrator and to familiarize them with the numbering of the vibrators (1 to 6). During the test, the vibrators were activated randomly for 50 ms, each stimulus repeated at least three times. The four bars in the stacked bar plots correspond to the four activated vibrators, divided into two groups for the left and right arm. The stacks are the percentage of answers for each perceived vibration: proximal, middle and distal. The percentage of middle vibrators corresponds to the percentage of error in

localizing the activated vibrator as the middle vibrator was never activated. Patients were able to localize the vibration successfully with percentages of 88% and 87% for proximal and distal vibrator localization. There were no discrimination errors between distal and proximal vibrators.

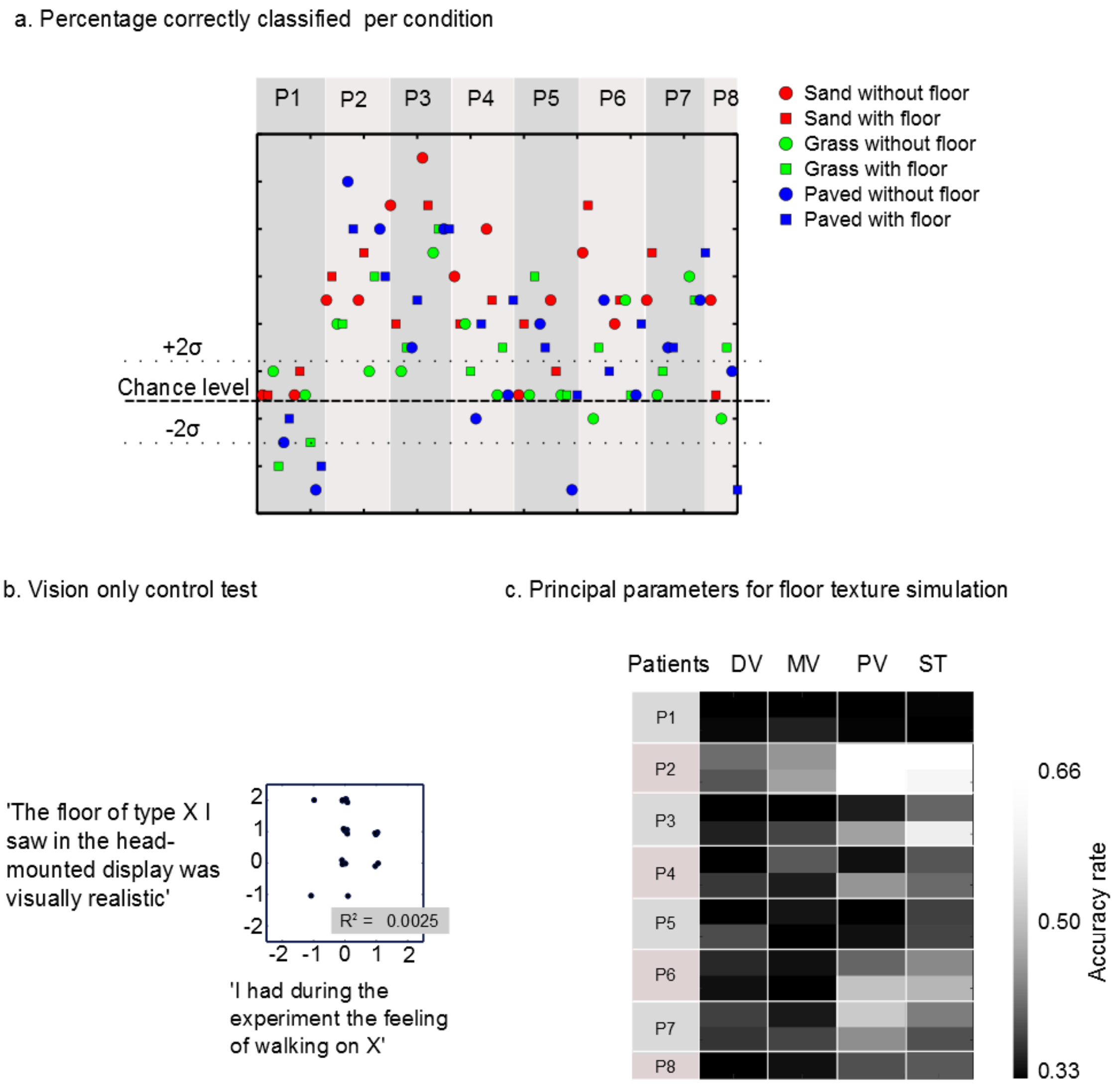

Fig S4. (**A**) Detail of patients accuracy per floor type during inverse task. (**B**) Subject's answer to Q2 (floor of type X I saw in the head mounted display was visually realistic) vs. rating of floor realism (only considering the realism of the virtual environment). No correlation was found between the responses to the two questions. (**C**) Principal tactile parameters considered by patients to discriminate the different floor texture is calculated using knn classifier on the 120 repetitions during exploratory phase (Chance level = 0.33). Classifier accuracy is given for all 15 sessions (row) and all parameters (columns). Parameters PV and ST are found to be best ones for all patients.

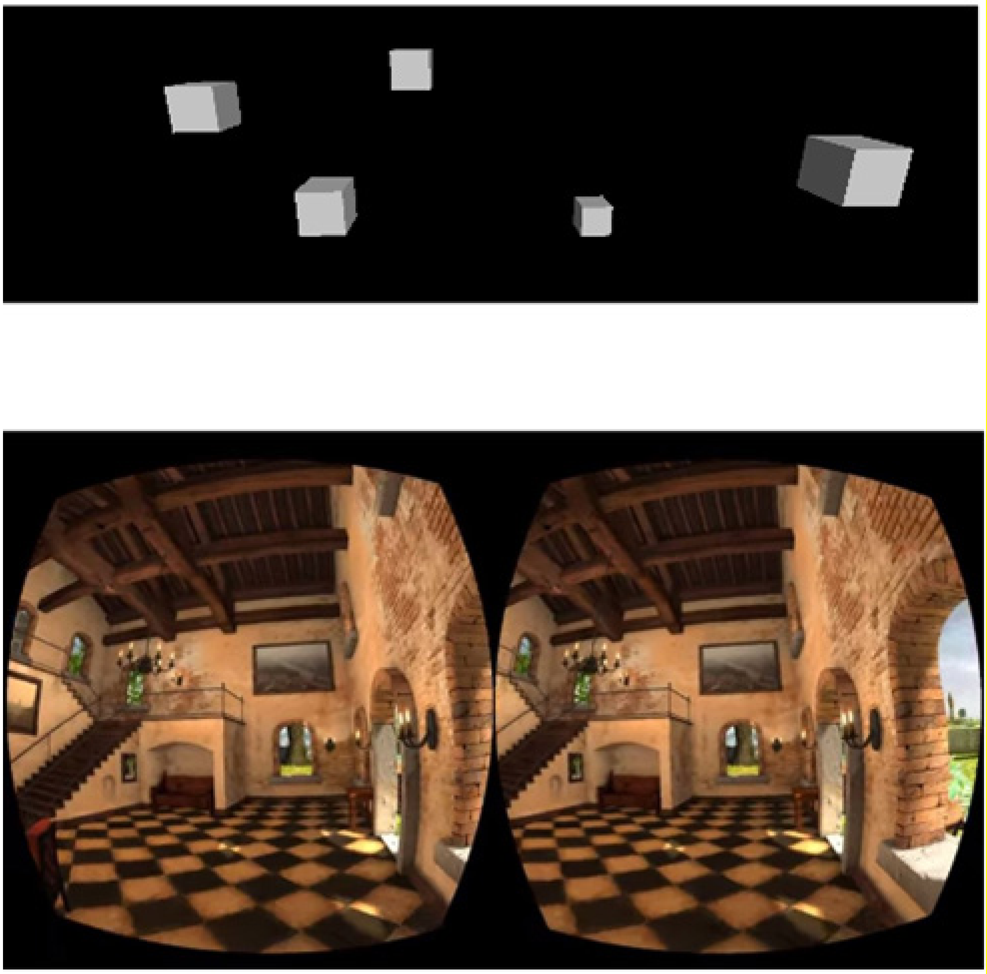

Fig. S5. Visual characterization. All patients had normal vision. We first evaluated patients' perception of 3D objects in the VR. A clinical condition referred to as stereo-blindness results in poor depth perception. A 3D world with 13 randomly sized and placed cubes were presented in the head mounted display. Patients had to move their head and find the cubes and describe their relative position to each other. All patients successfully passed the test. The second test evaluated patients' susceptibility to motion sickness. They had to use the keyboard and move their head to respectively translate and rotate the view in a simulated house (simulation of the Tuscany house https://share.oculus.com/app/oculus-tuscany-demo).

Table S2

	Gen.	Age	Lesion grade	Lesion level	Lesion Time (years)	Height (m)	Weight (kg)	Etiology
**Patient 1**	F	32	ASIA A	T 11 R<br>T 10 L	13	1.71	68	Traumatic
**Patient 2**	M	26	ASIA B	T 4 R<br>T 4 L	6	1.7	84	Traumatic
**Patient 3**	M	32	ASIA A	T 10 R<br>T 11 L	5	1.64	64	Traumatic
**Patient 4**	M	38	ASIA A	T 8 R<br>T 8 L	5	1.73	69	Traumatic
**Patient 5**	M	36	ASIA A	T 7 R<br>T 7 L	3	1.73	69	Traumatic
**Patient 6**	M	29	ASIA A	T 4 R<br>T 4 L	8	1.67	58	Traumatic
**Patient 7**	M	27	ASIA A	T 7 R<br>T 5 L	6	1,71	58	Traumatic
**Patient 8**	F	29	ASIA A	T 11 R<br>T 11 L	11	1,53	52	Traumatic

Table S1. Patients' demography

Table S2

	Stance , DtP			Stance, PtD		
Patient	50 step/min	66 step/min	100 step/min	50 step/min	66 step/min	100 step/min
**PA1**	0	0	0	0	3	0
**PA2**	1	3	1	0	3	1
**PA3**	3	3	3	2	3	3
**PA4**	2	2	3	1	1	1
**PA5**	3	3	3	2	2	2
**PA6**	0	3	3	0	0	0
**PA7**	3	3	3	3	3	3
**Average**	1.71	2.43	2.29	1.14	2.14	1.43
**Grand average**	2.14			1.57		

	Swing, DtP			Swing, PtD		
Patient	50 step/min	66 step/min	100 step/min	50 step/min	66 step/min	100 step/min
**PA1**	3	3	3	1	3	0
**PA2**	0	3	3	3	3	3
**PA3**	1	3	3	2	1	1
**PA4**	3	3	3	3	3	3
**PA5**	1	1	1	0	0	3
**PA6**	0	0	0	0	3	0
**PA7**	3	3	3	2	3	1
**Average**	1.57	2.29	2.29	1.57	2.29	1.57
**Grand average**	2.05			1.70		

Table S2. Detail of patients score for pseudo-proprioception test. Score is given for patients 1-7 for three speeds: 50, 66 and 100 steps per minute and for four tactile paradigms: feedback on stance or swing and direction of stimulation from Distal to Proximal (DtP) or Proximal to Distal (PtD). Relying on tactile feedback only, a score of 3 meant that subject was able to perfectly tell the position of the legs of the avatar. A score of 2 means correct positioning with a systematic contralateral de-phasing. A score of 1 means that subject confuses

the position during periods of changing of speed. A 0 score was given when a patient had systematic error on all phases of the task.
